# *Ab initio* investigation of CaO-ZnO alloys under high pressure

**DOI:** 10.1038/srep11003

**Published:** 2015-07-17

**Authors:** Xiaojing Sha, Fubo Tian, Da Li, Defang Duan, Binhua Chu, Yunxian Liu, Bingbing Liu, Tian Cui

**Affiliations:** 1 State Key Laboratory of Superhard Materials, College of physics, Jilin University, Changchun, 130012, P. R. China

## Abstract

Ca_*x*_Zn_1–*x*_O alloys are potential candidates to achieve wide band-gap, which might significantly promote the band gap engineering and heterojunction design. We performed a crystal structure search for CaO-ZnO system under pressure, using an *ab initio* evolutionary algorithm implemented in the USPEX code. Four stable ordered Ca_*x*_Zn_1–*x*_O structures are found in the pressure range of 8.7–60 GPa. We further constructed the pressure vs. composition phase diagram of CaO-ZnO alloys based on the detailed enthalpy calculations. With the increase in Ca concentration, the CaO-ZnO alloy first undergoes a hexagonal to monoclinic transition, and then transforms back to a hexagonal phase. At Above 9 GPa, there is no cubic structure in the alloys, in contrast to the insostructural components (B1-B1). The band gap of the Ca_*x*_Zn_1–*x*_O alloy shows an almost linear increase as a function of the Ca concentration. We also investigated the variation regularity of the band gap under pressure.

In the past several years, ZnO has been considered as an attractive material, given its unique physical properties, such as a wide direct band gap of 3.37 eV and high exciton binding energy (60 meV) at room temperature^1^. These properties make it suitable for a wide variety of applications, such as ultraviolet (UV) emittion and detection, surface acoustic wave (SAW) devices, gas sensors and transparent conducting electrodes^2^,^3^. In these desired applications, high-efficient ZnO-based light-emitting devices are particularly important. Mixing ZnO-based semiconductor alloys with other materials which possess even wider band gaps, allows for the fabrication of quantum wells and superlattices^4^,^5^. Alloying is an effective approach to fine-tune the band gap in the range of blue-green and ultraviolet wavelengths, which greatly promotes the band-gap engineering and heterojunction design^6^,^7^. For different desired band gaps, there are several candidates, such as MgO, BeO, and CaO^8^,^9^,^10^. The MgZnO alloy, which has high solubility, has been widely investigated^11^,^12^,^13^,^14^,^15^,^16^,^17^. However, phase segregation between ZnO and MgO was observed for Mg concentrations x ≥ 0.36 due to the different crystal structures and large lattice mismatch between ZnO and MgO. Specifically, MgO is cubic and ZnO is hexagonal. BeO (*E*_*g*_ = 10.6 eV) is another good alternative, and it has the same crystal structure as ZnO. However, at increased pressures, the BeO remains in the hexagonal wurtzite structure, and ZnO transforms into cubic structure at ~9 GPa. In the past years, some theoretical researches have been done to seek a stable BeZnO alloy^18^,^19^,^20^,^21^,^22^,^23^, yet not fully successful because the large difference in the ionic radii of Be and Zn usually causes the structure of BeZnO thermodynamically unstable.

CaO is an IIa-IVa compound with a rock-salt structure and a wide band-gap of 7.2 eV^24^,^25^, and Ca_*x*_Zn_1–*x*_O alloys are also candidates for wider band-gap materials. A number of first-principle calculations on Ca_*x*_Zn_1–*x*_O with wurtzite structures have been performed^26^,^27^,^28^, however, no stable structure has yet been found. Previous theoretical studies focused on creating alloys in a wurtzite structure by replacing Zn with Ca. For instance, the existence of metastable states at Ca concentrations of *x* = 0.25, 0.50 and 0.75 has been shown, however, these kinds of consequences are not stable. Bulk ZnO prefers the hexagonal wurtzite structure (B4) under ambient pressure and transforms into a cubic rocksalt structure (B1) at 9 GPa^1^, while CaO is in a rocksalt structure under 60 GPa. Generally, there is a large difference in the crystal structure between wurtzite hexagonal ZnO (B4) and rock salt cubic CaO (B1) that can cause unstable phase-mixing, as shown by Nazir’s *et al.*^26^. In this paper, we investigate the stable structures of Ca_*x*_Zn_1–*x*_O alloy under high pressure using first principles calculations. We examine the stability, structural and electronic properties of the Ca_*x*_Zn_1–*x*_O alloys with different Ca concentrations. These structural and electronic properties are crucial for hetero-structural design and optimized growth of the related quantum wells and superlattices.

## Results and discussion

**Figure 1:**
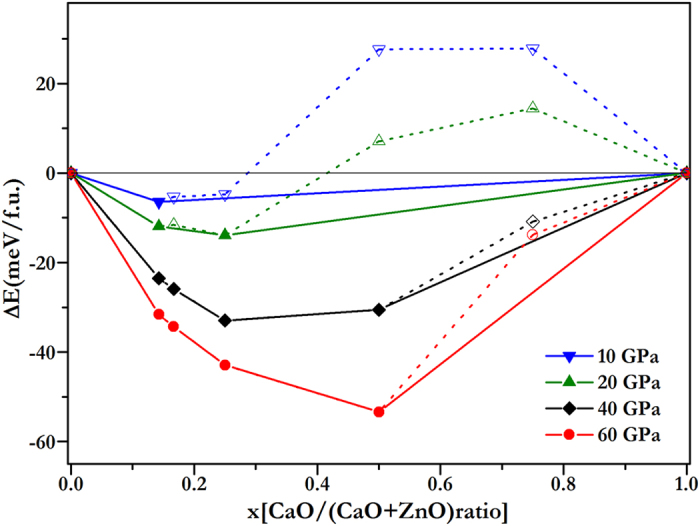
Convex hull diagram for CaO-ZnO alloys. The formation enthalpies (ΔH, with respect to ZnO and CaO of their most stable phases at selected pressures) of Ca_*x*_Zn_1−*x*_O. The abscissa *x* is the fraction of CaO in the structures. Circles on the solid lines represent stable ground-state compounds under the corresponding pressure.

**Figure 2:**
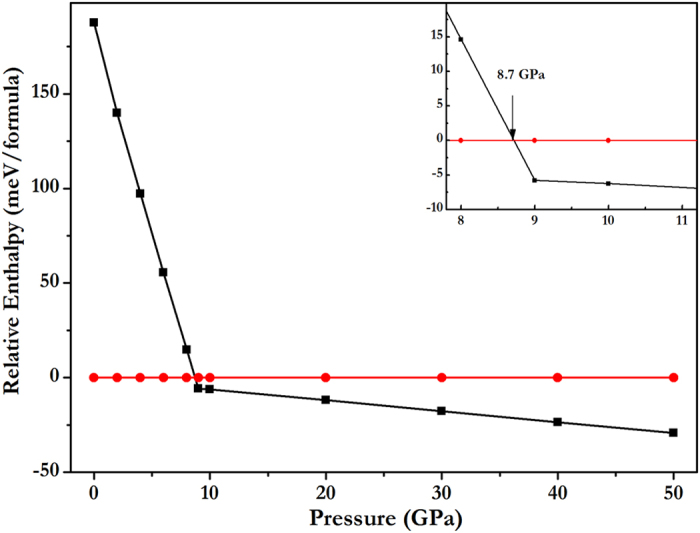
Enthalpy curves for R−3_CaZn_6_O_7_. (relative to ZnO and CaO in their most stable phases at selected pressures).

In this work, we report four stable ground state structures of Ca_*x*_Zn_1–*x*_O. The formation enthalpy of Ca_*x*_Zn_1–*x*_O alloy is calculated using a fractional representation of Ca_*x*_Zn_1–*x*_O (0 ≤ *x* ≤ 1) with respect to its decomposition into CaO and ZnO, as
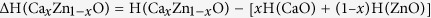
where x is the concentration of CaO. B1−CaO transits to B2 at 58 GPa in our calculation, similar to previous results^24^,^29^. In the condition of ZnO, the transition from B4 to B1 takes place at 9 GPa in our calculation, consistent with the experimental findings *P*_*t*_ ≈ 8.7 GPa^30^, *P*_*t*_ ≈ 9.1 GPa^31^, or *P*_*t*_ ≈ 10 GPa^32^ and first-principles calculations *P*_*t*_ = 8.8 GPa^12^. Thus, when pressure is below 9 GPa, B4 phases are considered, and above 9 GPa B1-ZnO is considered for the lowest energy. Similarly in the case of CaO, B1 phases below 58 GPa, and above 58 GPa, B2 phases are considered. The relative energetics of CaO-ZnO alloys from 10 to 60 GPa are summarized in the convex hull plots shown in Fig. 1. The formation enthalpies were evaluated as the difference in the enthalpy of the predicted CaO-ZnO alloys with respect to CaO and ZnO at the selected pressures. Structures lying on the convex hull are either thermodynamically stable or metastable, and can be synthesized in principle. Figure 1 reveals that CaZn_6_O_7_ is the first stable alloy below 10 GPa, which has the most negative enthalpy of formation. In Fig. 2 we show the calculated enthalpy difference of CaZn_6_O_7_ as a function of pressure, calculated against the decomposition into the constituent oxides (B1-CaO, B1-ZnO, or B4-ZnO). As shown in Fig. 2, the structure of CaZn_6_O_7_ becomes stable above 8.7 GPa. The space group of this high-pressure phase is R-3. In the case of B4-ZnO and B1-CaO, the formation enthalpies of all Ca_*x*_Zn_1–*x*_O alloys are positive before 8.7 GPa, indicating the tendency for the segregation into ZnO and CaO.

**Figure 3:**
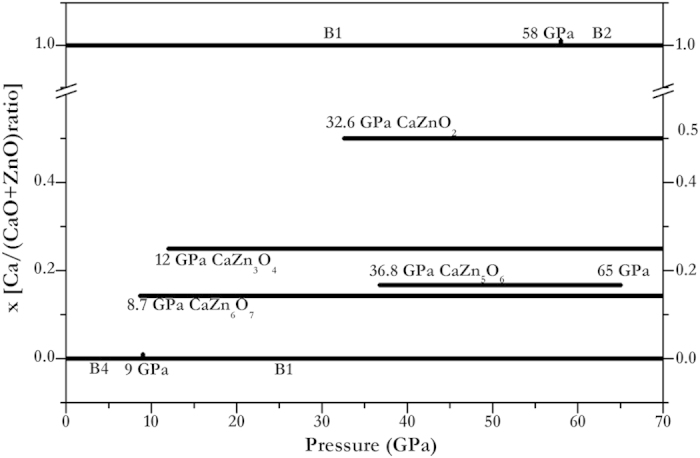
Pressure-composition phase diagram of the Ca_x_Zn_1−x_O alloys.

At above 8.7 GPa, more stable Ca_*x*_Zn_1–*x*_O structures appear. As depicted in Supplementary Fig. S1, enthalpy calculations reveal that CaZn_3_O_4_ alloys exist above 12 GPa. At higher pressure, CaZnO_2_ and CaZn_5_O_6_ become stable at 32.6 GPa and 36.8 GPa, respectively. The thermodynamic results show that the formation enthalpy of CaZn_5_O_6_ is always slight in the pressure range of 36.8–65 GPa. The CaZn_5_O_6_ structure will become unstable completely when pressure reaches 65 GPa, with a tendency to segregate into CaZn_6_O_7_ and CaZn_3_O_4_. All of the expected stable alloys of CaZn_6_O_7_ (*R–*3), CaZn_5_O_6_ (*C*2/*M*), CaZn_3_O_4_ (*P*2/*C*), and CaZnO_2_ (*R*–3*M*) are depicted in Fig. 3. More alloy structures with higher Ca-concentration appear with further increase in pressure. However, the structures can only be stable when the Ca-concentration is no more than 50%, even if pressure is increased over 60 GPa.

These detailed calculations for stable structures allow us to construct a P-*x* phase diagram of CaO-ZnO alloys, as shown in Fig. 3. There is a tendency for the number of Ca_*x*_Zn_1–x**_O alloys to increase with pressure, similar to Mg_*x*_Zn_1–x**_O alloys^12^. However, with pressure increasing, more Mg-rich structures appeared in the MgO-ZnO case, but more Zn-rich structures appear in our study. CaO, ZnO, and MgO all exhibit cubic structures under pressure; and the ionic radii of Ca, Zn and Mg is 1, 0.74, and 0.72 Å, respectively^33^. In this case, the element with the smaller ionic radius trend to turn into higher component in the alloys.

**Figure 4:**
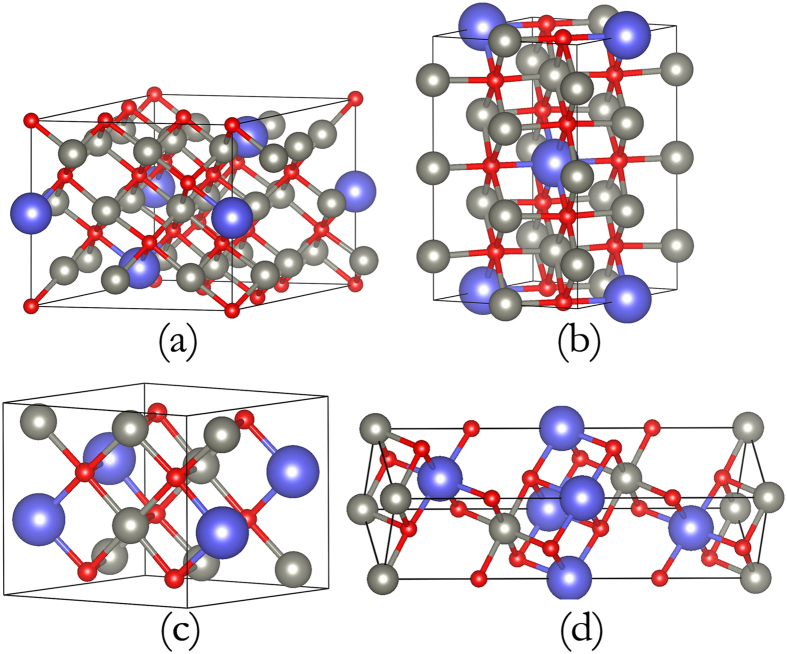
Crystal structures of the predicted Ca_x_Zn_1−x_O at the selected pressures. (**a**) R-3_CaZn_6_O_7_ at 40 GPa, (**b**) C2/m_CaZn_5_O_6_ at 60 GPa, (**c**) P2/c_CaZn_3_O_4_ at 40 GPa and (**d**) R-3 m_CaZnO_2_ at 40 GPa. The large purple, medium-sized gray and small red spheres represent Ca, Zn and O atoms, respectively. More structural details are listed in Table 1.

For all the predicted structures shown in Fig. 4, the corresponding structural information is listed in Table 1. We calculated the phonon dispersions under pressure, and found them to be dynamically stable (Supplementary Fig. S2). We also calculated the phonon dispersions and elastic constants of the four predicted structures under 0 GPa, as shown in Supplementary Fig. S3 and Table S1, which show the structures are mechanically and dynamically stable at 0 GPa. So we think our predicted alloys can be synthesized at high pressure and reserved at ambient conditions.

Previous studies suggest that the symmetry of component structures is important in alloys of ZnO with other group-II metal oxides^6^,^18^,^26^,^27^,^34^. Most of these structures were created using the substitution method, where Zn was replaced with Ca in the wurtzite supercell or Ca was replaced with Zn in the rocksalt supercell. However, our current study shows that the stable CaO-ZnO alloys undergo a transition from hexagonal to monoclinic, and back to hexagonal structure, with the increase in the Ca concentration. At 8.7 to 9 GPa, the CaZn_6_O_7_ alloy made from the nonisostructural components (wurtzite + rocksalt) is stable through both dynamics and thermodynamic analysis. At above 9 GPa, more structure became stable, however, none of the alloys have the rocksalt structure, without even the cubic symmetry adopted, although they were made from the isostructural components (rocksalt + rocksalt). In our previous study, we found similar results in the MgO-ZnO alloys^12^, where the alloys have a hexagonal structure with a high Mg-concentration, while both ZnO and MgO have a similar cubic structure under pressure. In BeO-ZnO alloys, the solubility of Be_*x*_Zn_1–x**_O in the Zn-rich region is low, although ZnO and BeO have the same wurtzite structure^18^. In addition, no BeO-ZnO alloy structures with thermodynamic stability were found using our USPEX calculations. In contrast to previous views, the symmetry of component structure shows little impacts on the alloy structure here.

**Figure 5:**
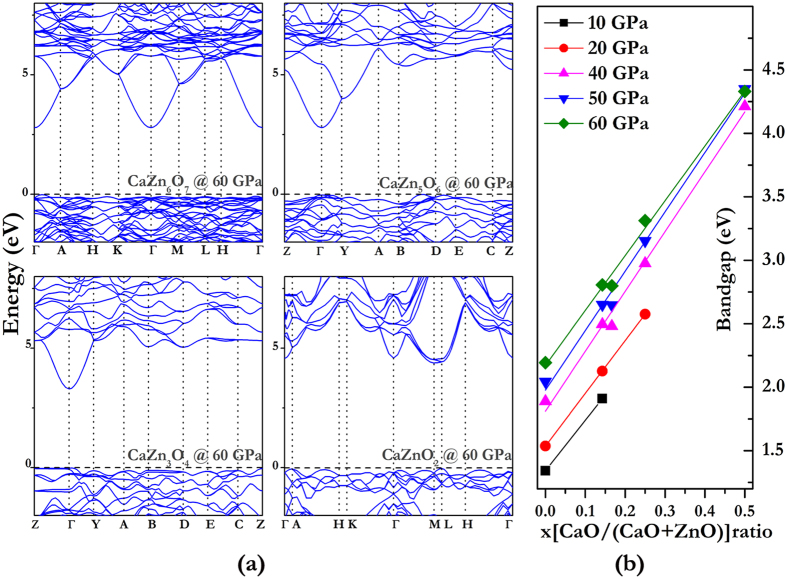
The electronic band structures of CaO-ZnO alloys. (**a**) Depicts the electronic band structures of CaZn_6_O_7_, CaZn_5_O_6_, CaZn_3_O_4_, CaZnO_2_ at 60 GPa. (**b**) Depicts the band gaps as the function of pressure at selected pressures.

Band gap (*E*_*g*_), which denotes the required energy of an electron from valence band maximum (VBM) to conduction band minimum (CBM), is an important parameter to determine the optoelectronic properties of semiconductors. The band gap of ZnO increases with pressure, and that of CaO decreases with pressure, as shown in Supplementary Fig. S3. Therefore, it is interesting to observe the pressure effects on band gaps of CaO-ZnO alloys. These band gaps are graphed in Fig 5(b) as a function of Ca composition for ground-state Ca_*x*_Zn_1–x**_O structures under high pressure. The band gap increases with pressure and also increases in an almost linear fashion with Ca concentration, when the pressure increases from 10 to 60 GPa. The more CaO mixing with ZnO, the wider band gap of alloy will be formed. In our previous theoretical study^12^, we also found the band gap linearly increases with Mg content in the Mg_x_Zn_1–x_O alloys, which is consistent with the experimental results^35^.

As shown in Fig. 5(b), band gaps of all alloys increase with pressure except CaZnO_2_, whose band gaps have different variation rules for pressure. Calculated electronic band structures are graphed in Fig. 5(a) at selected pressures. The CBMs of CaZn_6_O_7_, CaZn_5_O_6_ and CaZn_3_O_4_ are all located at the Γ point (0,0,0), and the CBM of CaZnO_2_ is located at the M point (0,1/2,0). The CBM transforms from the Γ point to the M point as the Ca concentration increases.

**Figure 6:**
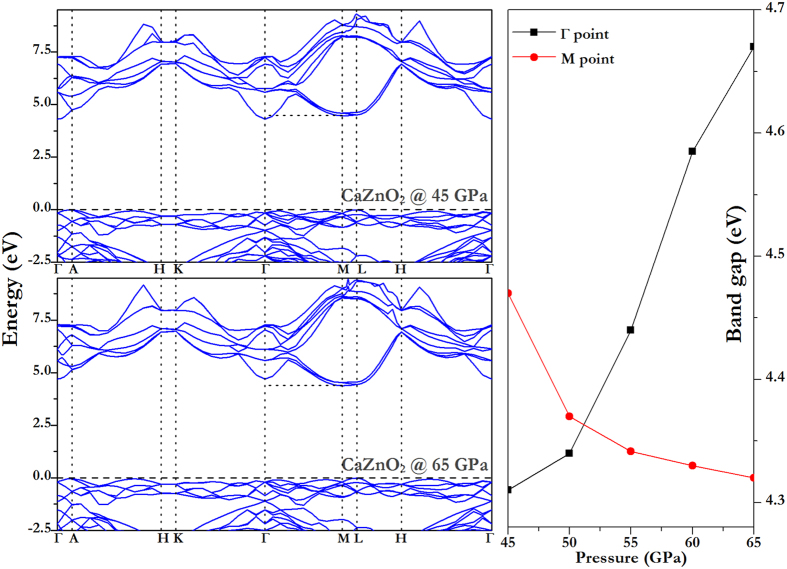
The electronic band structures of CaZnO_2_. At pressure of (**a**) 45 GPa, (**b**) 65 GPa, respectively. And (**c**) the band gap at the Γ and M points as the function of pressure are depicted by black and red lines, respectively.

We further analyze whether the different concentrations of alloys lead to a variation in the electronic band structures. Firstly, when Ca concentration is less than 0.5, the band gap increases with pressure, and the CBM is always located at the Γ point. Band gaps are plotted as a function of pressure in Supplementary Fig. S4. However, CaZnO_2_ displays some extraordinary properties. The band gap of CaZnO_2_ first increases and then decreases with pressure, as shown in Fig. 6. The CBM of CaZnO_2_ under 40 GPa is located at the Γ point (0, 0, 0), and the band gap at Γ point increases with pressure, similar to the behavior in B1-ZnO. Meanwhile, the CBM of CaZnO_2_ from 40 to 60 GPa is located at the M point (0, 1/2, 0), and the gap at the M point reduces slowly with pressure, same to CaO at the X point (1/2, 0, 1/2). Using qualitative analysis, we keep the lattice constants unchanged, remove Ca or Zn atoms, respectively, and analyze the band structures of the broken crystal. The CBM of CaZnO_2_ without Ca atoms located at the Γ point, and without Zn locates at the M point, which reveals that the conduction band at the Γ point basically comes from ZnO, and at the M point comes from CaO. Thus, with the increase in pressure, the CaO band-gap is more significant than the ZnO one.

**Figure 7:**
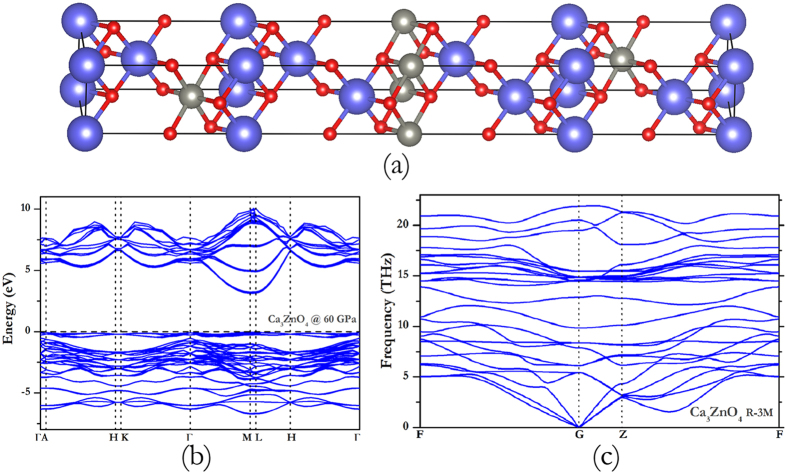
The metastable phase of Ca_3_ZnO_4_. (**a**) the crystal structure, (**b**) the electronic band structures and (**c**) the phonon dispersion curve.

Furthermore, other than the above-mentioned four stable structures, we find a metastable structure when Ca concentration is 0.75, as shown in Fig. 7(a). There is no imaginary frequency in Fig. 7(c), so the Ca_3_ZnO_4_ structure is dynamically stable. Although the formation enthalpy (with respect to ZnO and CaO) is negative as shown in Fig. 1 above 40 GPa, the enthalpy respect to CaZnO_2_ and CaO is always positive, which indicates a tendency to segregate into CaZnO_2_ and CaO. From the calculated electronic band structures in Fig. 7(b), the CBM of Ca_3_ZnO_4_ alloy is located at M the point. The band gap of this metastable structure is represented by the purple dotted line in Supplementary Fig. S4. The band gap of those large Ca-concentration structure decreases with pressure, while the low Ca-concentration structure exhibit the opposite behavior. In view of the band structure of all of these alloys, and considering the effects of pressure on band gap, the effect of Ca-concentration is also significant. In summary, the band gap of CaO-ZnO alloy depends on a fine interplay between concentration and pressure.

## Conclusion

We investigate the composition and structure of the CaO-ZnO alloys at high pressure using USPEX codes. In a range of 0–60 GPa, four stable alloy structures with different Ca concentrations have been found. With increasing pressure, more alloys with higher Ca concentrations appear. However, the structure can only be stable when the Ca-concentration is no more than 50%, with pressure up to 60 GPa. When the Ca concentration increases, the stable structures of CaZn_6_O_7_, CaZn_5_O_6_, CaZn_3_O_4_ and CaZnO_2_ undergo a hexagonal to monoclinic transition, and then transforms back to a hexagonal phase. We find that ground-state structures of the alloys do not share the cubic nature of ZnO and CaO, indicating the symmetry of component structures has little impact on the alloy structure. Through detailed analysis of band structure, we note an almost linear increase in band gap as a function of Ca-concentration at the selected pressure. When the concentration is below 50% the band gap increases with pressure. The band gap of CaZnO_2_ first increases with pressure up to 40 GPa, and then starts to decrease with pressure.

## Methods

To search for the stable and low-enthalpy structures of CaO-ZnO alloy, an *ab initio* evolutionary algorithm (EA), which has been designed to find the most stable structure for a given chemical composition range at given external conditions as implemented in the USPEX code, is employed^36^,^37^,^38^. In this work, evolutionary crystal structure prediction calculations were performed at 0, 20, 40 and 60 GPa for (CaO)_m_–(ZnO)_n_ system (m + n ≤ 20), all at zero temperature. We performed *ab initio* calculations with the Local Density Approximation (LDA)^39^ as implemented in the Vienna *ab initio* simulation package (VASP) code^40^, which is based on density functional theory. The electron-ion interaction was described by the projector augmented wave (PAW) scheme^41^. The electron configurations 10*d*2*p*, 6*p*2*s* and 4*p*2*s* were treated as valence for Zn, Ca, and O, respectively. During structural relaxation, a tested energy cut off of 550 eV was used for the plane wave basis sets, and a k-point resolution of 0.03 Å^–1^ in the reciprocal space was used for all structures to minimize error from the k-point meshes. The atomic positions, lattice parameters, and cell volume were fully relaxed until the force on each atom was less than 1 meV/Å. Phonons were calculated with the supercell method implemented in the PHONOPY program.

## Additional Information

**How to cite this article**: Sha, X. *et al.* *Ab initio* investigation of CaO-ZnO alloys under high pressure. *Sci. Rep.* **5**, 11003; doi: 10.1038/srep11003 (2015).

## Supplementary Material

Supplementary Information

## Figures and Tables

**Table 1:** Lattice constants, formation enthalpy, atomic coordinates, and pressure for CaO-ZnO alloys.

	**Structure**	**Parameters (Å,°)**	**Atom**	**x**	**y**	**z**	**P (GPa)**
CaZn_6_O_7_	R-3 (Hexagonal axes)	a = b = 7.6251	Ca(3b)	0.33333	0.66667	0.16667	40
		c = 7.0743	Zn(18f)	0.61726	0.09667	0.16741	
			O(3a)	0.0	0.0	0.0	
			O(18f)	0.08623	0.46419	0.34274	
CaZn_5_O_6_	C2/M (unique axis b)	a = 5.0062	Ca(2c)	0.0	0.0	0.5	60
		b = 8.6712	Zn(4g)	0.0	0.16565	0.0	
		c = 5.0020	Zn(4h)	0.0	0.33317	0.5	
		β = 70.6589	Zn(2b)	0.0	0.5	0.0	
			O(8j)	0.26194	0.8246	0.24489	
			O(4i)	0.2613	0.0	0.76382	
CaZn_3_O_4_	P2/C (unique axis b)	a = 5.8219	Ca(2e)	0.0	0.36660	0.75	40
		b = 5.8332	Zn(2e)	0.0	0.87625	0.75	
		c = 5.0617	Zn(2f)	0.5	0.37016	0.75	
		β = 125.8119	Zn(2f)	0.5	0.87512	0.75	
			O(4g)	0.74649	0.11137	0.76219	
			O(4g)	0.27035	0.36636	0.27462	
CaZnO_2_	R-3M (Hexagonal axes)	a = b = 3.0269	Ca(3b)	0.66667	0.33333	0.83333	40
		c = 13.9889	Zn(3a)	0.33333	0.66667	0.66667	
			O(6c)	0.0	0.0	0.74247	
