# Supplementary material for: Ab initio investigation of CaO-ZnO alloys under high pressure
Source: Sci Rep. 2015 Jul 17;5:11003. doi: 10.1038/srep11003 (PMC4505315; doi:10.1038/srep11003)

Supplementary information

Ab initio investigation of CaO-ZnO alloys under high pressure

Xiaojing Sha, Fubo Tian, Da Li, Defang Duan, Binhua Chu, Yunxian Liu, Bingbing Liu and Tian Cui*

State Key Laboratory of Superhard Materials, College of Physics, Jilin University, Changchun 130012, People's Republic of China.

**Corresponding author, E-mail: cuitian@jlu.edu.cn*

Table SI The elastic constants of CaO-ZnO alloys at 0 GPa

$CaZn_6O_7$	C_{11}	C_{33}	C_{44}	C_{12}	C_{13}	C_{66}	
	2604.84	2507.19	615.79	971.65	1042.12	666.78	
$CaZnO_2$	C_{11}	C_{33}	C_{44}	C_{12}	C_{13}	C_{66}	
	2447.79	2129.39	637.15	985.91	1177.12	554.66	
$CaZn_3O_4$	C_{11}	C_{22}	C_{33}	C_{44}	C_{55}	C_{66}	C_{12}
	2540.35	2328.14	2511.01	832.28	871.11	391.58	720.75
	C_{13}	C_{15}	C_{23}	C_{25}	C_{35}	C_{46}	
	1266.71	-39.94	1336.71	-32.89	6.42	12.19	
$CaZn_5O_6$	C_{11}	C_{22}	C_{33}	C_{44}	C_{55}	C_{66}	C_{12}
	2534.01	2382.55	2626.84	547.03	620.52	674.35	1005.73
	C_{13}	C_{15}	C_{23}	C_{25}	C_{35}	C_{46}	
	855.89	123.45	828.06	-258.77	-80.40	-241.17	

Figure S1 Calculated enthalpies as the function of pressure. (a) Enthalpy curves (relative to $CaZn_6O_7$ and CaO) for $CaZn_3O_4$, (b) Enthalpy curves (relative to $CaZn_3O_4$ and CaO) for $CaZnO_2$, (b) Enthalpy curves (relative to $CaZn_6O_7$ and $CaZn_3O_4$) for $CaZn_5O_6$, (e) Enthalpy curves (relative to $CaZnO_2$ and CaO) for Ca_3ZnO_4.

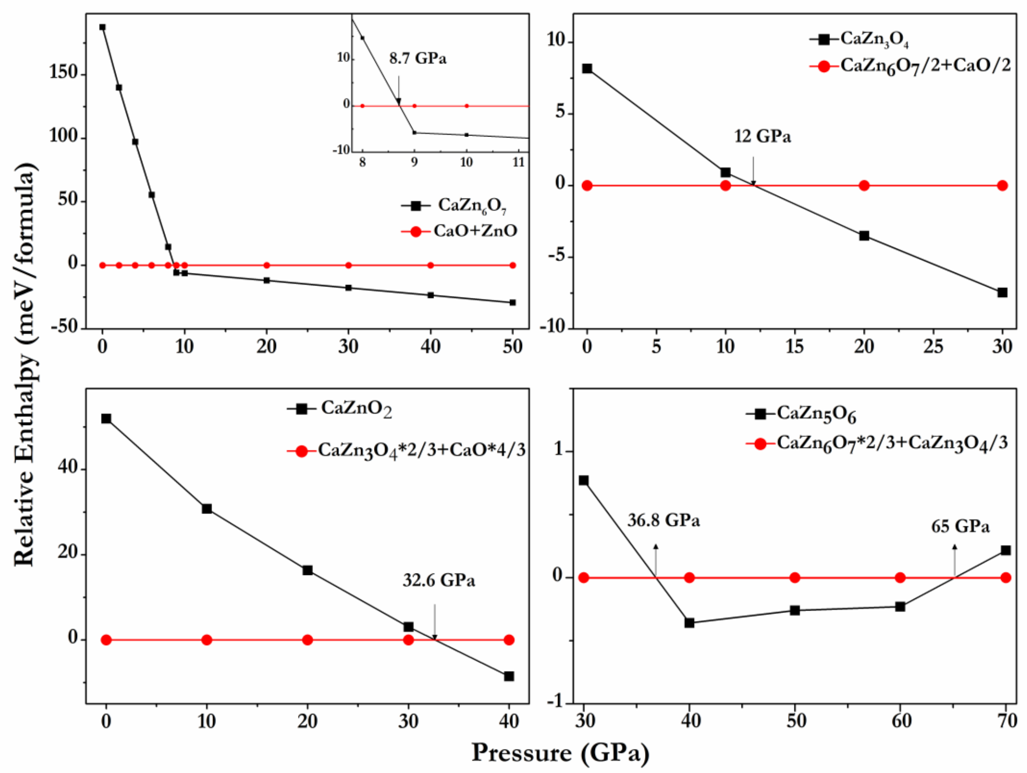

Figure S2 Dynamics stability of new CaO-ZnO alloys. The phonon dispersion curves for (a) R-3_$CaZn_6O_7$, (b) C2/m_$CaZn_5O_6$, (c) P2/c_$CaZn_3O_4$, (d)R-3m_$CaZnO_2$, (e)R-3m_Ca_3ZnO_4, and (f) P-1_$CaZn_4O_5$ at selected pressures.

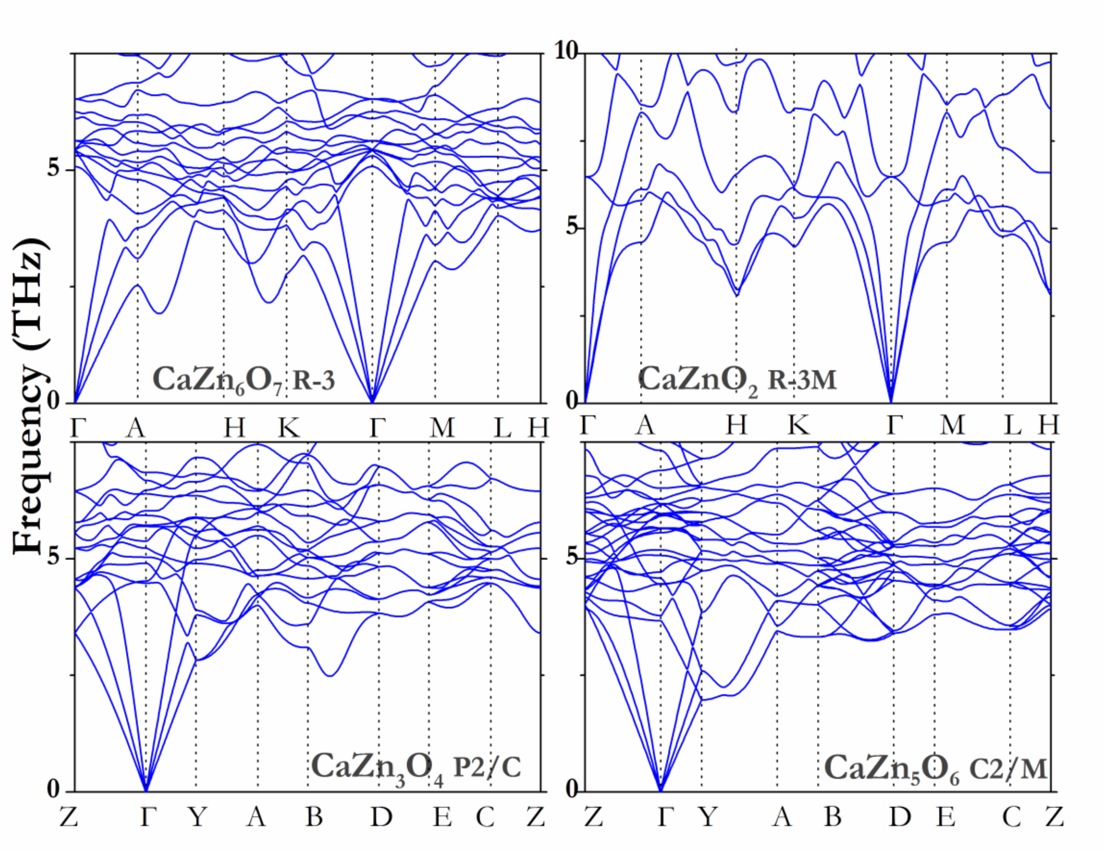

Figure S3 Dynamics stability of new CaO-ZnO alloys. The phonon dispersion curves for (a) R-3_$CaZn_6O_7$, (b) C2/m_$CaZn_5O_6$, (c) P2/c_$CaZn_3O_4$, (d)R-3m_$CaZnO_2$, (e)R-3m_Ca_3ZnO_4, and (f) P-1_$CaZn_4O_5$ at 0 GPa.

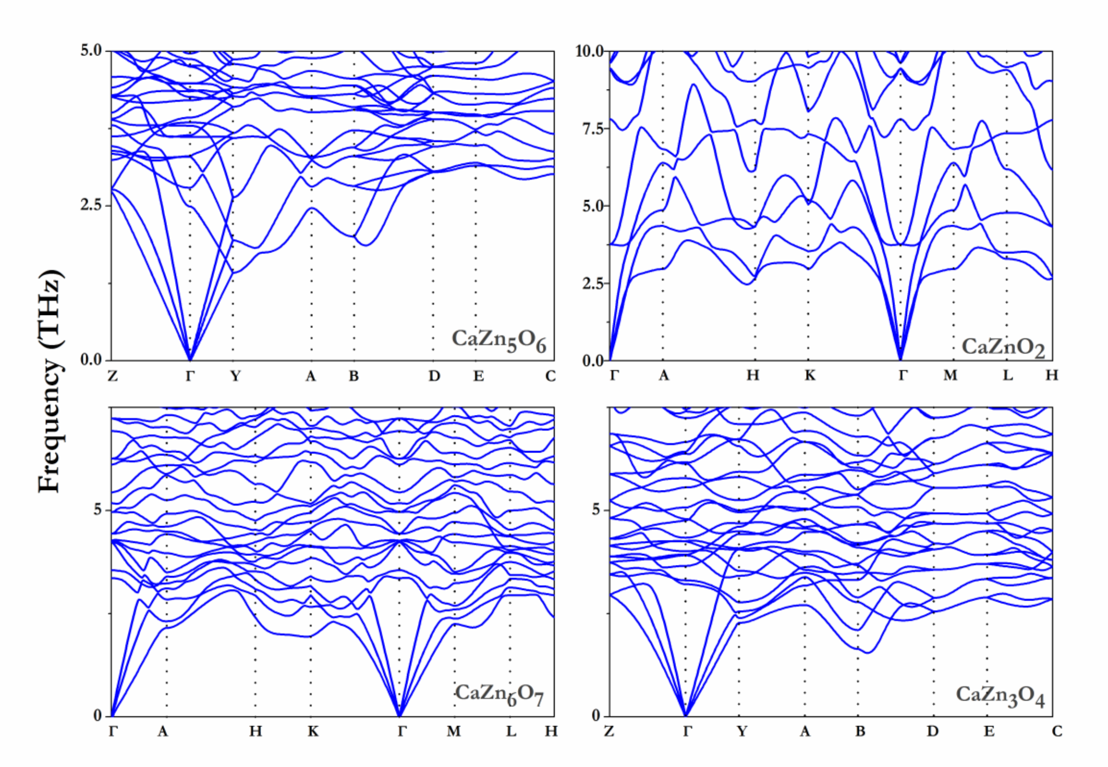

Figure S4 The band gap of CaO and ZnO as the function of pressure depicted in purple and blue lines, respectively.

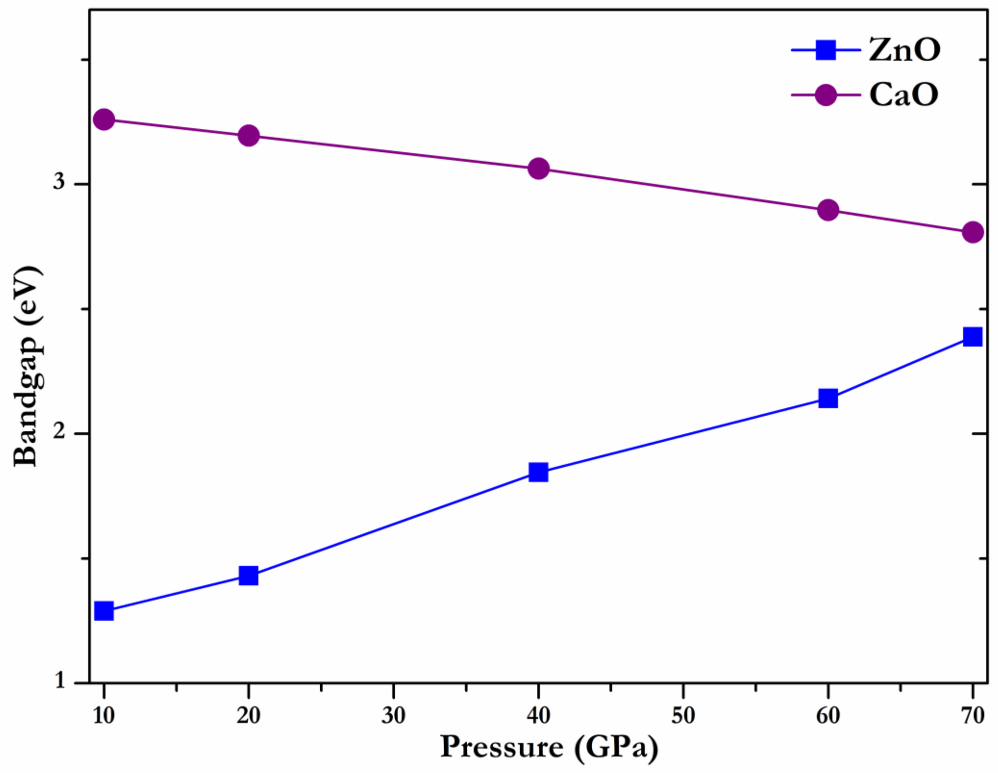

Figure S5 The band gap of structures with different Ca concentration as the function of pressure. Band gap of stable structures is given by the solid lines, and one of metastable structures is given by the dotted lines.

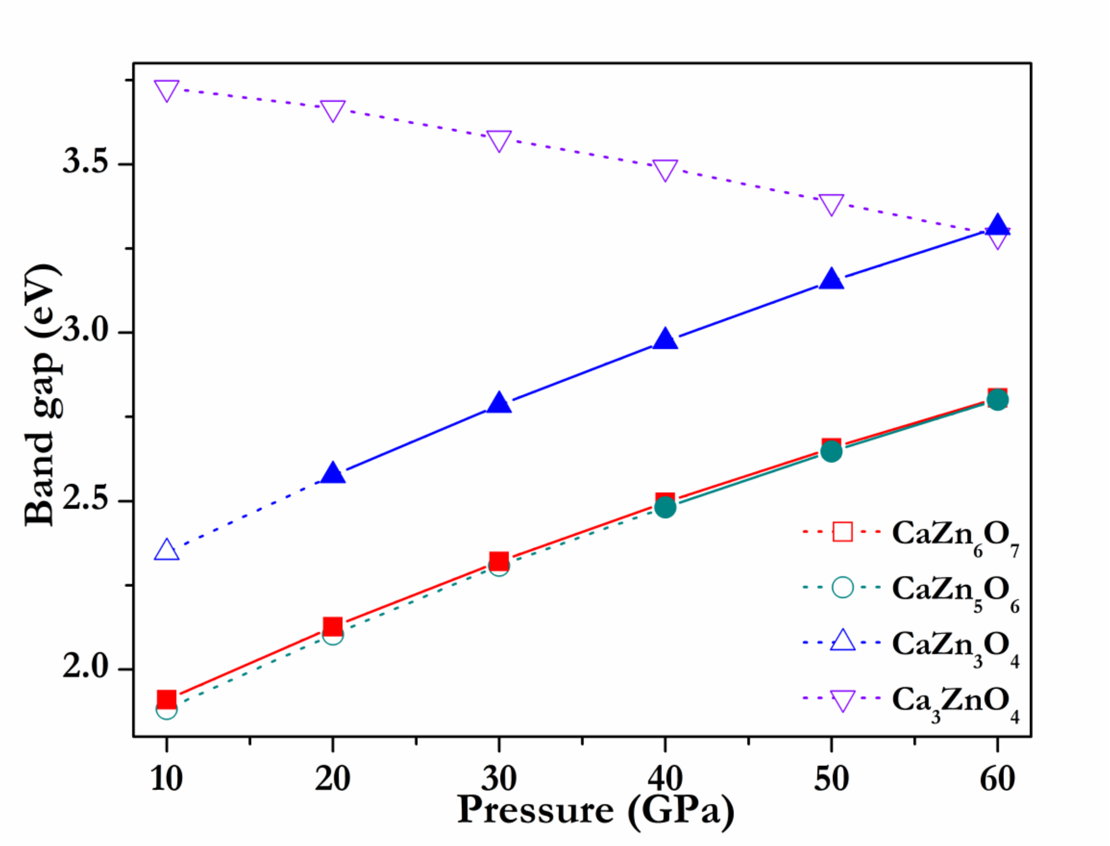

Supplement: Supplementary Information [file srep11003-s1.pdf]
